# Low MSP-1 haplotype diversity in the West Palearctic population of the avian malaria parasite *Plasmodium relictum*

**DOI:** 10.1186/s12936-021-03799-8

**Published:** 2021-06-12

**Authors:** Olof Hellgren, Victor Kelbskopf, Vincenzo A. Ellis, Arif Ciloglu, Mélanie Duc, Xi Huang, Ricardo J. Lopes, Vanessa A. Mata, Sargis A. Aghayan, Abdullah Inci, Sergei V. Drovetski

**Affiliations:** 1 Department of Biology, Lund University, Lund, Sweden; 2 Department of Parasitology, Faculty of Veterinary Medicine, Erciyes University, Kayseri, Turkey; 3 Vectors and Vector-Borne Diseases Implementation and Research Center, Erciyes University, Kayseri, Turkey; 4 Nature Research Centre, Akademijos 2, 08412 Vilnius, Lithuania; 5 MOE Key Laboratory for Biodiversity Sciences and Ecological Engineering, Beijing Normal University, Beijing, China; 6 CIBIO, Centro de Investigação Em Biodiversidade E Recursos Genéticos, InBIO Laboratório Associado, Universidade Do Porto, Vairão, Portugal; 7 Yerevan State University, 1 Alex Manoogian, Yerevan, 0025 Republic of Armenia; 8 US Geological Survey, Eastern Ecological Research Center at Patuxent Research Refuge, Beltsville, MD 20705 USA; 9 Present Address: Department of Entomology and Wildlife Ecology, University of Delaware, Newark, DE USA

**Keywords:** Avian malaria, Haemosporida, Host specificity, Merozoite surface protein 1, Parasite population structure, *Plasmodium relictum*

## Abstract

**Background:**

Although avian *Plasmodium* species are widespread and common across the globe, limited data exist on how genetically variable their populations are. Here, the hypothesis that the avian blood parasite *Plasmodium relictum* exhibits very low genetic diversity in its Western Palearctic transmission area (from Morocco to Sweden in the north and Transcaucasia in the east) was tested.

**Methods:**

The genetic diversity of *Plasmodium relictum* was investigated by sequencing a portion (block 14) of the fast-evolving merozoite surface protein 1 (MSP1) gene in 75 different *P. relictum* infections from 36 host species. Furthermore, the full-length MSP1 sequences representing the common block 14 allele was sequenced in order to investigate if additional variation could be found outside block 14.

**Results:**

The majority (72 of 75) of the sequenced infections shared the same MSP1 allele. This common allele has previously been found to be the dominant allele transmitted in Europe.

**Conclusion:**

The results corroborate earlier findings derived from a limited dataset that the globally transmitted malaria parasite *P*. *relictum* exhibits very low genetic diversity in its Western Palearctic transmission area. This is likely the result of a recent introduction event or a selective sweep.

## Background

The amount of standing genetic variation in a pathogen population can affect its ability to adapt to changes in its host population or environment. It also reflects the pathogen’s population history: timing, trajectory and rate of past population fluctuations [1, 2]. If a genetic variant in a host–pathogen system is strongly favoured by selection, it is likely to rapidly increase in frequency, reducing the genetic diversity of that locus in the population. This, however, will have no effect on the genetic diversity of other pathogen populations where the novel variant may not exist or where selection does not favour it. Similarly, patterns of low genetic variation (across the genome) in a pathogen population might stem from an introduction event (founder effect) followed by rapid expansion in a host or vector population. While a founder effect is likely to result in low genetic diversity across the genome, selection for a particular allele would likely result in low diversity at a single locus and linked loci only. Thus, patterns of genetic variation in a pathogen population can elucidate whether selection has occurred, the rate of a new mutation becoming fixed, and whether there has been a recent introduction event into a new area or host population [3–6]. Furthermore, population genetic variation can be used to infer the geographical origin of pathogen populations [3, 5]

In contrast to the small number of malaria species infecting humans, avian *Plasmodium* parasites, commonly referred to as avian malaria parasites, are much more speciose [7–9]. Many decades ago, avian malaria was used as a model study system to better understand human malaria biology. However, the mouse malaria model soon replaced avian malaria as a model system [10, 11]. In recent years, avian malaria re-emerged as a model system for research into ecology and evolution of wildlife parasites [12]. While it previously has been difficult to sequence nuclear genes of these parasites, genome and transcriptome sequences are becoming increasingly available with the development of modern sequencing technologies [13–18]. These new data allow for investigations of intra- and interspecific genetic variation of avian malaria parasites at a higher resolution than was previously possible.

*Plasmodium relictum* is one of the most widespread avian malaria parasites [19–21] and is highly virulent in immunologically naïve avian species inhabiting remote islands and zoological gardens [22, 23]. The morphologically defined *P. relictum* consists of several mitochondrial cytochrome b (cyt b) lineages [19–21]. These lineages appear to be associated with different transmission areas. For example, the lineage SGS1 is primarily transmitted within tropical and temperate regions of the Old World whereas the lineage GRW4 occurs globally, although its transmission is limited to areas with warmer climates [21]. Furthermore, *P. relictum* cyt b lineages differ in evolutionary independence. SGS1 and GRW11, for example, share nuclear haplotypes in some populations likely suggesting a common recent evolutionary history, or that they belong to the same recombining population. In contrast, neither SGS1 nor GRW11 share nuclear haplotypes with GRW4, which points to the independent evolutionary history of GRW4 and SGS1/GRW11 [21].

The fast-evolving nuclear merozoite surface protein 1 (*msp1*) gene provides a more fine-scale genetic resolution of the parasite lineages than does the mitochondrial cyt b gene [24]. The *msp1* gene is involved in the invasion of host’s red blood cells and a part of the gene codes for a protein segment that is exposed to the host immune system. This exposure is thought to lead to its relatively fast evolutionary rate likely due to selection to avoid detection. As a result, high *msp1* allelic diversity has been found in natural populations of other *Plasmodium* species [25, 26]. However, studies of *P. relictum* found a remarkably low haplotype diversity of the *msp1* gene in SGS1 and GRW11 lineages transmitted in Europe; nevertheless, *msp1* diversity appeared higher in vectors engorged with passerine blood in Japan [21]. These findings raised the possibility that the *P. relictum* lineages colonized Europe from other areas, possibly Asia, where haplotype diversity of parasites appears higher, at least in vectors [21]. However, Hellgren et al. [21] analysed data from only five avian species, three of which were from the taxonomic family Passeridae. This limited host species sampling might have been the reason that low MSP1 diversity was found in Europe.

This study, significantly expands Hellgren et al. [21] dataset of *P. relictum msp1* sequences by adding 27 host species sampled across the Western Palearctic in order to address: (1) whether the originally discovered low *msp1* haplotype diversity of SGS1 and GRW11 in the Western Palearctic resulted from limited sampling or, alternatively, was in fact low; and, (2) if the diversity is high in the expanded dataset, whether there is an association between host species and parasite *msp1* haplotypes, or if *msp1* haplotypes are randomly distributed among host species. If the diversity of *msp1* haplotypes is higher than originally estimated, the hypothesis is that selection imposed by different host immune systems should result in the non-random distribution of *msp1* haplotypes among host species. This potential cryptic specialization of *msp1* alleles may have been missed in studies using the slower evolving cyt b gene for lineage identification. Alternatively, the lack of *msp1* haplotype structure among host species and its extremely low haplotype diversity would be consistent with previous work and suggest a recent population expansion of the parasites into the Western Palearctic.

## Methods

Genetic diversity at the *msp1* gene was analysed in two sample sets using two different sequencing methodologies. For the first, larger sample set, block 14 of the *msp1* gene (249 bp out of 269 bp) was amplified and sequenced with Sanger sequencing. In order to investigate if there was variation in the *msp1* gene that was missed by just sequencing block 14 (the complete *msp1* gene is 4740 bp in length), the complete MSP1 gene was sequenced in a smaller sample set using a sequence capture method. The rationale behind this is that the paper by Hellgren et al. [24], where the complete *msp1* gene of *P. relictum* was sequenced, relied on a PCR protocols, that are not nested as the block 14 protocol, using samples with extremely high parasitaemias obtained through infection experiments; such parasitaemia levels are almost never observed in the wild. Therefore, amplifying the full *msp1* gene from samples obtained from natural infections using PCR and Sanger sequencing is more or less impossible. One way to circumvent this problem and to obtain full-length sequence data of nuclear genes is to use sequence capture methods [27].

### Amplification and analysis of *msp1* block 14

Block 14 of the *msp1* gene was amplified from 27 avian blood samples infected with the *P. relictum* cyt b lineage GRW11 and 111 samples infected with cyt b lineage SGS1 using the primers and annealing temperatures described in Hellgren et al. [21] and sequenced with Sanger methodology (in-house). Note that the name of the internal primer pair, MSP1_3FN and MSP1_3RN, have been named incorrectly regarding their direction to the outside primers, MSP1_3F and MSP1_3R in the original article [21]. These samples were part of previous studies [28, 29], which identified single haemosporidian infections with one of the two *Plasmodium* cyt b lineages. All samples were collected under legal permits. These samples originated from 36 passerine species captured at 35 localities in Portugal, Morocco, Russia, and Armenia (Table 2). Blood was sampled by brachial venipuncture with a sterile needle and collected into a heparin-free glass capillary tube. Samples were immediately transferred into 2-ml vials with 96% ethanol and stored until DNA extraction as described previously [21].

New *msp1* block 14 sequences (GenBank; Pr10: MZ270634 and Pr11: MZ270635) were aligned to those from Hellgren et al. [21] using Geneious Prime 2019.2.3. (www.geneious.com). The phylogenetic relationships among new and previously discovered [21] *msp1* haplotypes were evaluated by Bayesian phylogenetic analysis, using MrBayes [30] as implemented in Geneious Prime 2019.2.3. Phylogenies was constructed using 1,100,000 iterations and sampling every 200th tree under a general time reversal + invariable site model (GTR + I) allowing for six different substitution rates. After discarding 10% of the sampled trees as a burn-in, the remaining trees were used to construct a majority consensus tree. The phylogeny contained all known *msp1* haplotypes originating from cyt b SGS1 and GRW11 infections. As outgroups (for outgroup sequences see [21]) *msp1* haplotypes Pr4 (KJ850282) and Pr5 (KJ850281) originating from the cyt b lineage GRW4 was used. For visualization of the relationship and frequency of obtained haplotypes a haplotype network which was inferred using PopArt version 1.7.2 [31], with the TCS inference method [32] was constructed.

### Sequence capture of *msp1*

For an additional 23 samples, collected under legal permits, with SGS1 (n = 20) or GRW11 (n = 3) infections confirmed using the molecular screening described in Hellgren et al. [33], the complete *msp1* gene was sequenced following the sequence capture protocol described in Huang et al. [18], but using a probe set designed to target *P. relictum* instead (available upon request) after the presence of SGS1 (n = 20) or GRW11 (n = 3) infection was confirmed using the molecular screening protocol described in Hellgren et al. [33]. Capture probes targeting 1,036 genes from the *P. relictum* genome [15] was constructed and used in a SureSelectXT Target Enrichment kit (Agilent Technologies) as previously described [18], with a few modifications. The protocol started with 200 ng of sheared DNA from each sample and followed the manufacturer’s protocol for sequence capture, including hybridizing probes with the samples for 24 h. For the PCR, 18 cycles of was used in the post-capture amplification with indexed primers (instead of the recommended 10 to 16 cycles) to ensure sufficient amount of DNA for sequencing. Captured fragments were sequenced on an Illumina MiSeq instrument at the Lund University DNA Sequencing Facility.

Raw sequencing reads was processed by removing adapter sequences and low-quality base calls with a sliding window using Trimmomatic v0.39 [34]. Read quality was assessed with FastQC v0.11.9. The reads were mapped to the *P. relictum* genome (PlasmoDB.org, release 39 [35]) using Nextgenmap v0.5.5 [36] with default options. The resulting sam files were compressed, sorted and indexed using samtools v1.10 [37]. MarkDuplicates v2.20.8 in Picard tools (Broad Institute, 2019) was used to identify duplicate reads. To ensure that the infections were indeed single infections and matched SGS1 or GRW11 cyt b sequences, the consensus sequence was first checked against the SGS1 and GRW11 reference sequences [9]. Then double infections were identified by visually detecting SNPs in the mapped reads of the cyt b gene. For individuals that showed no evidence of double infections at the cyt b gene, the majority consensus sequences of the *msp1* gene were generated for sites with a depth of coverage > 5 as implemented in Geneious Prime 2019.2.3. Discarding double infections left 11 samples, of which five had incomplete coverage; these 11 samples were used for further analysis at different degrees. The full-length consensus sequences were first aligned against each other using the MUSCLE alignment algorithm with eight iterations in Geneious Prime 2019.2.3. This allowed the identification of SNPs outside the *msp1* block 14 section (targeted in the larger sample set). Next, the full-length *msp1* genes were mapped against the shorter reference sequences of *msp1* block 14, found in Hellgren et al. [21], to assess the haplotype identity of the complete *msp1* sequences. Those previously identified *msp1*haplotypes of *P. relictum* have been named Pr1-Pr9 [21] and will be referred to as such thereafter; novel haplotypes were given numbers > Pr9, numbered in the order in which they were discovered.

## Results

### *msp1* block 14

Block 14 of the *msp1* gene was successfully amplified and sequenced in 11 samples of GRW11 originating from nine avian species and in 64 samples of SGS1 from 25 avian species sampled in Russia, Armenia, Morocco, and Portugal (GenBank accession numbers MZ270578-MZ270624). Haplotype Pr2 was discovered in 72 of these 75 infections. Samples that were not sequenced failed in amplification of the nuclear gene, a common problem when trying to sequence nuclear genes of avian malaria due to low parasitaemia in wild hosts [27]. A single adult common whitethroat (*Sylvia communis*) infected with GRW11 from Armenia had the Pr3 haplotype, which previously had been found only in vectors caught and sampled in the Eastern Palearctic, i.e., Japan [21]. The remaining two haplotypes were new and both found in SGS1 infections. Pr10 was found in an adult rock-thrush (*Monticola saxatilis*) from Armenia and Pr11 was found in a juvenile Cetti’s warbler (*Cettia cetti*) from Portugal (Table 2). The majority of the Pr2 haplotypes linked to both GRW11 and SGS1 infections were found in resident bird species confirming their local transmission in the sampling areas.

**Fig. 1:**
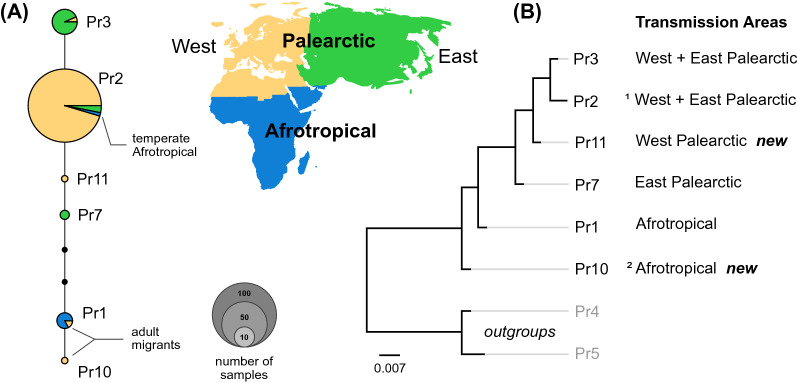
**A** Haplotype network of MSP1 haplotypes found in *Plasmodium relictum* (cytochrome b lineages SGS1 and GRW11). Each circle represents a haplotype with the size proportional to its sampling frequency. Colours represent the area where the infected bird was sampled, not taking into account whether it was a migratory species or if it was a juvenile before its first migration or an adult. Black dots on lines represent the number of additional mutational steps between two haplotypes. **B** Phylogenetic reconstruction of the MSP1 haplotypes found in the SGS1 and GRW11 cyt b lineages of the *Plasmodium relictum.* Transmission area is determined if the haplotype have been recovered from juvenile or resident birds in an area. Scale bar represents nucleotide substitutions per site. ^1^ Pr2 has also been found in two cases in a non-migratory bulbul species (*Pycnonotus capensis*)endemic to a region in South-Africa with a Mediterranean climate. Pr10 and Pr11 are new haplotypes identified in this study. ^2^ Pr10 has only been found in a single adult African-migratory bird in Armenia and is therefore likely transmitted in the Afrotropical region

The phylogenetic reconstruction and the haplotype network placed the Pr11 haplotype together with other haplotypes transmitted either in the Western Palearctic (Pr2) or in the Eastern Palearctic (Fig. 1). In fact, the Pr11 haplotype only differed by a single synonymous substitution from Pr2, the dominant lineage transmitted in the Western Palearctic. The other new haplotype, Pr10, found in an adult Paleotropical migrant (*Monticola saxatilis*) was a sister to the SGS1/GRW11 clade and differed by a single non-synonymous substitution from Pr1, which has Afrotropical transmission (Fig. 1).

### *msp1* sequence capture

After discarding samples that showed evidence of double infections at the cyt b gene, 11 samples remained and are referred to in the subsequent analysis. The complete *msp1* gene, 4740 bp, was sequenced with a coverage depth > 5 in two GRW11 and three SGS1 infected samples. For the remaining six samples, 0.2–43.4% of the nucleotide positions had a coverage depth < 5 × and were excluded from the analysis (Table 3). All five complete samples were unambiguously assigned to the haplotype Pr2 at the MSP1 block 14 and all consensus sequences where monomorphic across the rest of the gene, showing no genetic variation across these samples (represented by GenBank entry MW385258). However, all of them (GenBank accession numbers MZ270625-MZ270635) differed by a single substitution from the reference sequence (KC969175) on GenBank but were identical to the *msp1* gene in the genome of the SGS1 parasite available at PlasmoDB [15, 35]. This discrepancy may point to a potential sequencing error in the reference sequence KC969175 [24].

## Discussion

The results confirm previous work that documented very low genetic diversity of the avian blood parasite *P. relictum* in its Western Palearctic transmission area [21]. The lack of diversity suggests that a single variant of the pathogen may have gone through a bottleneck or a selective sweep in the Western Palearctic. Furthermore, this extremely low diversity was found at the usually highly diverse merozoite surface protein 1 (*msp1*) gene. Earlier work found a single *msp1* haplotype transmitted in Europe [21] in a very limited number of host species, i.e., house martins (*Delichon urbicum*), common crossbills (*Loxia Curvirostra*) and house sparrows (*Passer domesticus*) (Table 1). In this study the *msp1* haplotypes was identified in 75 *P. relictum* infections (11 GRW11 and 64 SGS1) from 36 different host species. The vast majority of these infections (72 of 75) had the same *msp1* haplotype (Pr2). Of the three remaining haplotypes found, −Pr11 was found in a non-migratory bird in Portugal that suggests its transmission in Europe. These results strengthen the picture that there is one major *msp1* haplotype that is transmitted in Europe and neighbouring temperate regions: in this study there were 72 cases (92%) of a single haplotype (Pr2), and only a single case of a haplotype (Pr11) other than the dominant one found in individual hosts that either belonged to resident bird species or juveniles caught before they had migrated to another area (Table 2).

**Table 1:** Summary of MSP1 (block 14) diversity in *Plasmodium relictum* cyt b lineages SGS1 and GRW11 found in previous studies

Host species	Number of cases	Lineage	Haplotype	Migration status	Country
*Acrocephalus arundinaceus*	1	GRW11	Pr2	Mig	Bulgaria
***Passer domesticus***	**1**	**GRW11**	**Pr2**	**Res**	**Bulgaria**
***Passer domesticus***	**3**	**GRW11**	**Pr2**	**Res**	**Italy**
Blood in vector	1	GRW11	Pr2	Res	Japan
Blood in vector	1	GRW11	Pr3	Res	Japan
Blood in vector	1	GRW11	Pr7	Res	Japan
***Passer domesticus***	**2**	**GRW11**	**Pr2**	**Res**	**Lithuania**
*Pycnonotus capensis*	1	GRW11	Pr2	Res	South Africa
***Passer domesticus***	**1**	**GRW11**	**Pr2**	**Res**	**Spain**
*Delichon urbicum* (adult)	1	SGS1	Pr1	Mig	Spain
***Delichon urbicum* (juveniles)**	**3**	**SGS1**	**Pr2**	**Mig**	**Spain**
*Delichon urbicum* (adults)	14	SGS1	Pr2	Mig	Spain
*Acrocephalus arundinaceus*	1	SGS1	P2	Mig	Bulgaria
***Passer domesticus***	**1**	**SGS1**	**Pr2**	**Res**	**Bulgaria**
***Passer domesticus***	**4**	**SGS1**	**Pr2**	**Res**	**Italy**
Blood in vector	3	SGS1	Pr2	Res	Japan
Blood in vector	13	SGS1	Pr3	Res	Japan
Blood in vector	1	SGS1	Pr7	Res	Japan
*Passer domesticus*	2	SGS1	Pr1	Res	Kenya
***Loxia curvirostra***	**2**	**SGS1**	**Pr2**	**Res**	**Lithuania**
***Passer montanus***	**1**	**SGS1**	**Pr2**	**Res**	**Lithuania**
*Cercotrichas galactotes*	1	SGS1	Pr1	Res	Nigeria
*Cercotrichas podobe*	1	SGS1	Pr1	Res	Nigeria
*Passer luteus*	1	SGS1	Pr1	Res	Nigeria
*Pycnonotus capensis*	1	SGS1	Pr2	Res	South Africa
***Passer domesticus***	**4**	**SGS1**	**Pr2**	**Res**	**Spain**
***Passer domesticus***	**3**	**SGS1**	**Pr2**	**Res**	**Turkey**

Cases where transmission could only have happened in Europe are highlighted in bold. Migration status refers to whether the bird is a long-distance migratory species (Mig) or a resident species (Res). Data compiled from Garcia-Longoria et al. [37] and Hellgren et al. [21]

**Table 2:** Host and sampling location of birds infected with *Plasmodium relictum* cyt b lineages SGS1 and GRW11 and the *msp1* haplotype of the infection

Host species	Number of cases	Lineage	Haplotype	Migration status	Age	Country
*Sylvia communis*	1	GRW11	Pr2	Mig	AD	Armenia
*Sylvia communis*	1	GRW11	Pr3	Mig	AD	Armenia
*Cyanistes teneriffae*	1	GRW11	Pr2	Res	AD	Morocco
*Fringilla coelebs*	1	GRW11	Pr2	Res	AD	Morocco
*Passer domesticus*	1	GRW11	Pr2	Res	JUV	Morocco
*Passer hispaniolensis*	1	GRW11	Pr2	Res	AD	Morocco
*Periparus ater*	1	GRW11	Pr2	Res	AD	Morocco
*Cyanistes caeruleus*	1	GRW11	Pr2	Res	JUV	Portugal
*Parus major*	1	GRW11	Pr2	Res	Unknown	Portugal
*Phoenicurus ochruros*	1	GRW11	Pr2	Mig	AD	Portugal
*Carpodacus erythrinus*	1	SGS1	Pr2	Mig	AD	Armenia
*Cettia cetti*	1	SGS1	Pr2	Res	AD	Armenia
*Emberiza calandra*	1	SGS1	Pr2	Mig	AD	Armenia
*Monticola saxatilis*	1	SGS1	**Pr10**	Mig	AD	Armenia
*Parus major*	1	SGS1	Pr2	Res	AD	Armenia
*Sylvia curruca*	1	SGS1	Pr2	Mig	AD	Armenia
*Certhia brachydactyla*	1	SGS1	Pr2	Res	AD	Morocco
*Chloris chloris*	1	SGS1	Pr2	Res	JUV	Morocco
*Cyanistes teneriffae*	1	SGS1	Pr2	Res	AD	Morocco
*Oenanthe oenanthe*	1	SGS1	Pr2	Mig	AD	Morocco
*Periparus ater*	1	SGS1	Pr2	Res	AD	Morocco
*Phoenicurus moussieri*	2	SGS1	Pr2	Res	AD	Morocco
*Sylvia borin*	1	SGS1	Pr2	Mig	AD	Morocco
*Carduelis carduelis*	1	SGS1	Pr2	Res	JUV	Portugal
*Certhia brachydactyla*	2	SGS1	Pr2	Res	JUV	Portugal
*Cettia cetti*	1	SGS1	**Pr11**	Res	JUV	Portugal
*Cettia cetti*	7	SGS1	Pr2	Res	JUV	Portugal
*Cyanistes caeruleus*	1	SGS1	Pr2	Res	AD	Portugal
*Cyanistes caeruleus*	2	SGS1	Pr2	Res	JUV	Portugal
*Emberiza cia*	2	SGS1	Pr2	Res	Unknown	Portugal
*Emberiza cirlus*	1	SGS1	Pr2	Res	JUV	Portugal
*Erithacus rubecula*	1	SGS1	Pr2	Res	Unknown	Portugal
*Fringilla coelebs*	1	SGS1	Pr2	Res	AD	Portugal
*Hippolais polyglotta*	1	SGS1	Pr2	Res	JUV	Portugal
*Lophophanes cristatus*	1	SGS1	Pr2	Res	Unknown	Portugal
*Parus major*	6	SGS1	Pr2	Res	AD	Portugal
*Parus major*	3	SGS1	Pr2	Res	JUV	Portugal
*Parus major*	1	SGS1	Pr2	Res	Unknown	Portugal
*Passer domesticus*	3	SGS1	Pr2	Res	AD	Portugal
*Periparus ater*	3	SGS1	Pr2	Res	AD	Portugal
*Periparus ater*	4	SGS1	Pr2	Res	JUV	Portugal
*Phoenicurus ochruros*	3	SGS1	Pr2	Mig	AD	Portugal
*Phoenicurus ochruros*	2	SGS1	Pr2	Mig	JUV	Portugal
*Sylvia atricapilla*	2	SGS1	Pr2	Res	JUV	Portugal
*Sylvia melanocephala*	1	SGS1	Pr2	Res	JUV	Portugal
*Muscicapa striata*	1	SGS1	Pr2	Mig	JUV	Russia
*Parus major*	1	SGS1	Pr2	Res	AD	Russia

Migration status refers to whether the bird is a long-distance migratory species (Mig) or a resident species (Res). Age refers to whether the bird was an adult (AD) or juvenile (JUV) at capture. New lineages (Pr10 and Pr11) are highlighted in bold

The other haplotypes that were found were Pr10, a haplotype closely related to those previously found being transmitted in Afrotropical avian species [21] or identified in adult migratory birds that have been captured in Europe while returning from tropical Africa. This haplotype was found in an adult rock-thrush in Armenia. Rock-thrushes migrate to tropical Africa and it is therefore likely that this haplotype’s origin is in tropical Africa since it has not been found in juveniles or resident bird species in the Western Palearctic. If Pr10 is transmitted in Africa only as suggested (and Pr11 is transmitted in Europe only), the phylogenetic tree of the haplotypes suggests a single jump to temperate zone transmission by the ancestor of haplotypes Pr2, Pr3, Pr11 and Pr7 (Fig. 1b).

In this study the haplotype Pr3 was found in an adult common whitethroat. This is a haplotype that sits in the temperate transmission cluster in the phylogeny (Fig. 1b) with only one non-synonymous nucleotide difference from the dominant Pr2 (Fig. 1a). The haplotype was sequenced from an infection of the cyt b lineage GRW11, a lineage that is thought to be transmitted exclusively in temperate regions [21]. The haplotype Pr3 has previously only been found in vectors that have taken blood meals from birds residing in Eastern Asia (the jungle crow *Corvus macrorhynchos* and Asian house martin *Delichon dasypus*) [21]. In no case has the Pr3 been detected in tropical wintering areas of the common whitethroats which suggests that the transmission in this case had likely taken place at the breeding grounds in Armenia.

Although the diversity of host species samples used for sequence capture was not high, the lack of genetic diversity across the entire *msp1* gene showed that the *msp1* diversity in this and previous studies by investigating only block 14 (269 bp) of the almost 5 kb long gene was not underestimated (Table 3).

**Table 3:** Host species and sampling location of infections of *Plasmodium relictum* cyt b lineages SGS1 and GRW11 used for sequencing the full *msp1* gene

Host species	Lineage	Haplotype	Sample location	% coverage less than 5x	Gaps in MSP1 block 14
*Coccothraustes coccothraustes*	GRW11	Pr2	Lithuania	4 (187)	0
*Passer domesticus*	GRW11	Pr2	Lithuania	0	0
*Passer domesticus*	GRW11	Pr2	Lithuania	0	0
*Passer domesticus*	SGS1	Pr2	Bulgaria	11.2 (530)	0
*Passer domesticus*	SGS1	Pr2	Bulgaria	43.4 (2056)	89/269 (32.5%)
*Passer domesticus*	SGS1	Pr2	Bulgaria	32.5 (1536)	14/269 (5.2%)
*Passer domesticus*	SGS1	Pr2	Lithuania	0	0
*Passer domesticus*	SGS1	Pr2	Lithuania	0	0
*Passer domesticus*	SGS1	Pr2	Lithuania	0.2 (8)	0
*Coccothraustes coccothraustes*	SGS1	Pr2	Lithuania	0	0
*Loxia curvirostra*	SGS1	Pr2	Lithuania	1.1 (50)	0

% coverage refers to the percentage of nucleotides, out of the total of  4737 bp (excluding the stop codon), that had a sequence coverage less than 5; in parenthesis the actual number of nucleotide positions are presented. Gaps in *msp1* block 14 refers to the number of nucleotide positions, out of the total, at this section of the gene, that had missing data (in parenthesis the % is presented)

## Conclusion

*Plasmodium relictum* is one of the most common avian malaria parasites across the globe [9, 21] and is a morphologically defined species consisting of a clade of cyt b lineages which show marked differences in distribution and transmission areas [21]. In order to understand how this parasite has come to inhabit almost every corner of the Earth and seems to have fine-scale genetic differentiation that is linked to differences in ecological limitations [9, 21], variation in virulence [38, 39], and transmission [21, 40], there is a need to accurately describe its population genetics and the relationships among genetic lineages. For the *P. relictum* parasites transmitted in Europe, this study confirms that there is little genetic variation at *msp1* throughout the parasite population. One possible explanation for the lack of genetic variation could be a recent introduction event into the temperate region, with reduced genetic variation as a result of a rapid population expansion. To fully understand the evolutionary history of this parasite there is a need to generate more multi-locus or genomic data, a task that has recently begun [13, 27], to document whether the low genetic diversity at *msp1* is reflective of the entire genome or perhaps a result of selection. There is also a need for more extensive sampling in tropical areas in order to contrast the genetic variation found in Europe with that found in other transmission areas. Comparisons of the population genetics of multiple parasite lineages that differ in ecological traits such as transmission area or host preference will ultimately provide the opportunity to uncover the genetic basis of such traits and potentially reveal the historical circumstances that led to lineage differentiation and pathogen dispersal.

## Data Availability

The datasets analysed in this study are available from the corresponding author on reasonable request.
